# How the Colourless ‘Nonfluorescent’ Chlorophyll Catabolites Rust

**DOI:** 10.1002/chem.201003313

**Published:** 2011-02-18

**Authors:** Markus Ulrich, Simone Moser, Thomas Müller, Bernhard Kräutler

**Affiliations:** [a] Institute of Organic Chemistry and Centre of Molecular Biosciences, University of InnsbruckInnrain 52a, 6020 Innsbruck (Austria)

**Keywords:** catabolites, chlorophyll, plant pigments, tetrapyrroles

For a long time, the appearance of the fall colours has been associated with the enigmatic chlorophyll breakdown in higher plants.[1] However, the extensive earlier search for coloured chlorophyll breakdown products has remained unsuccessful.[2] When chlorophyll catabolites from higher plants were first tentatively identified, they were indicated to be colourless.[3, 4] These colourless compounds readily decomposed to rust-coloured materials upon analysis by thin-layer chromatography and were thus named “rusty pigments”, originally.[3, 4] The puzzling picture cleared up, when one of the presumed chlorophyll breakdown products was structurally characterized as a colourless linear tetrapyrrole,[4] the type of which is meanwhile classified as a “nonfluorescent” chlorophyll catabolite (NCC, see Scheme 1).[2, 5] Indeed, the colourless NCCs are ubiquitous in various senescent leaves and have been considered to represent the major “final” products of chlorophyll breakdown in senescent plants.[6] However, *Cj-*NCC-1 (**1**), a colourless NCC isolated from senescent leaves of the deciduous tree *Cercidipyllum japonicum* (Katsura tree) could be chemically oxidized to a yellow chlorophyll catabolite, named *Cj*-YCC, which has also been detected in fall leaves recently.[7] Here, we analysed the major coloured products, when **1** decomposed to ‘rust’ on silica gel.

**Scheme 1:**
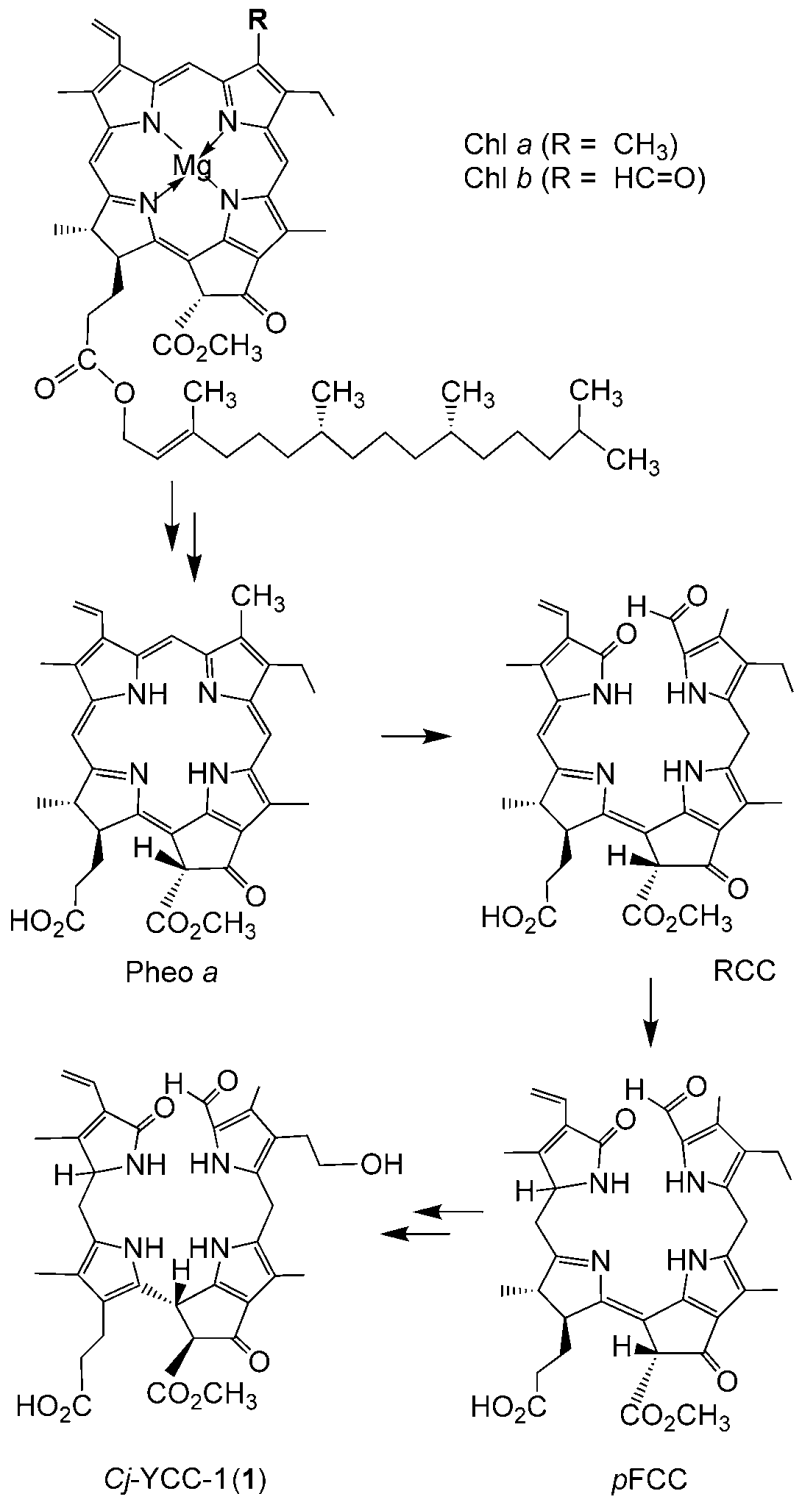
Structural outline of chlorophyll breakdown in senescent leaves: chlorophylls a and b are rapidly degraded to the colourless, nonfluorescent chlorophyll catabolites (NCCs), such as *Cj*-NCC-1 (**1**), an NCC found in leaves of *Cercidiphyllum japonicum*.[5, 8, 9]

Application of a solution of *Cj*-NCC-1 (**1**)[8, 9] to a silica gel TLC plate first gave a nearly colourless “spot”, which acquired a brown colour (“rust”) within 2–5 min, when exposed to air and daylight. TLC analysis of such a “spot” of **1** separated off a yellow zone on the plate, as was observed earlier with “rusty pigments”,[3, 4] and an additional pink-red spot developed eventually. Thin-layer re-chromatography in a second dimension of the TLC trace originating from the NCC **1** revealed the yellow fraction to directly form on the plate from the colourless **1**, whereas the pink-red spot correlated with the yellow fraction (see Figure S1 in the Supporting Information).

In an analytical experiment, NCC **1** (13.8 mg, 21.4 μmol) was adsorbed on silica gel 60 (5 g). The slightly yellow powder was suspended in hexane (20 mL) and was exposed to daylight, while being stirred magnetically under air. After 90 min the powder had acquired an orange-red colour, and it was extracted with MeOH. The orange-red extract contained a colourless fraction of **1** (*r*_t_=17 min), two yellow compounds (*Cj*-YCC-1 (**2 b**), *r*_t_=15 min; *Cj*-YCC-2 (**2 a**), *r*_t_=22 min) and a pink red fraction (*Cj*-PiCC (**3**), *r*_t_=35 min), as seen by analytical reversed-phase HPLC (see Figure 1). In a preparative experiment, NCC **1** (192.9 mg) was adsorbed on silica gel 60 (19.3 g). The dried powder was stirred magnetically under air, while being illuminated with a 100-W tungsten lamp. After 14 h the powder had acquired an orange-red colour. The adsorbed organic compounds were eluted with MeOH. The crude product mixture was separated by RP-MPLC. A colourless fraction of the NCC **1** (33.5 mg, 17.4 %), two main YCCs, **2 b** (29.2 mg, 15.2 %) and **2 a** (30.3 mg, 15.8 %), and the pink-red PiCC **3** (5.5 mg, 2.9 %) were isolated and were obtained as dry powders (see Experimental Section).

**Figure 1:**
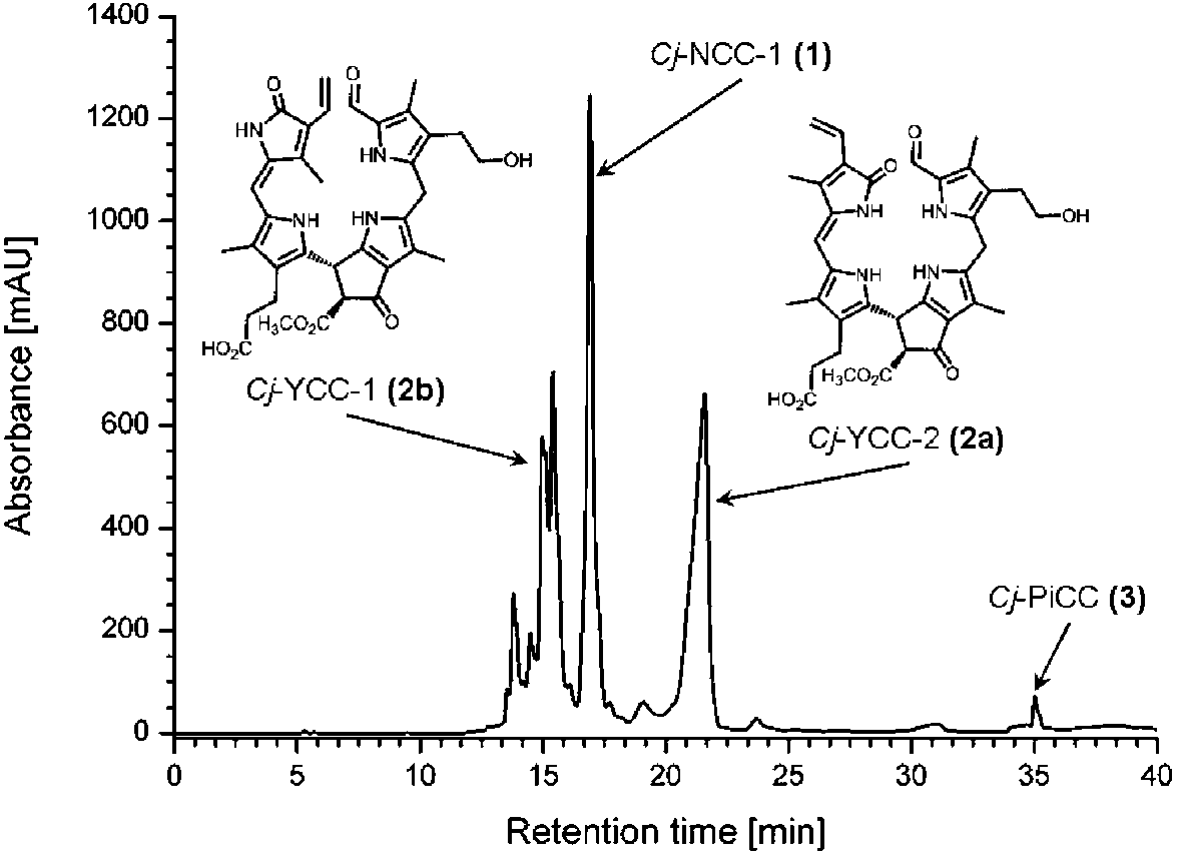
HPLC-analysis of the raw product mixture of the preparative oxidation of **1** on silica gel (detection at *λ*=320 nm), and structural formulae of *Cj*-YCC-1 (**2 b**) and *Cj*-YCC-2 (**2 a**).

*Cj*-YCC-2 (**2 a**), the less polar of the two yellow compounds, was obtained earlier by direct oxidation of *Cj*-NCC-1 (**1**) and was characterized as (13^2^*S*,15*R*,20*Z*)-3^1^,3^2^-didehydro-4,5,10,15-(22,24 *H*)hexahydro-13^2^-methoxycarbonyl-4,5-*seco*-4,5-dioxophytoporphyrinate (**2 a**, then tentatively named *Cj*-YCC, see Figure 1).[7] The UV/Vis spectrum of this yellow tetrapyrrole exhibited three characteristic maxima at 244, 310 and 426 nm (in methanol, relative intensities of 0.50:0.69:1.00, see Figure 2).[7]

**Figure 2:**
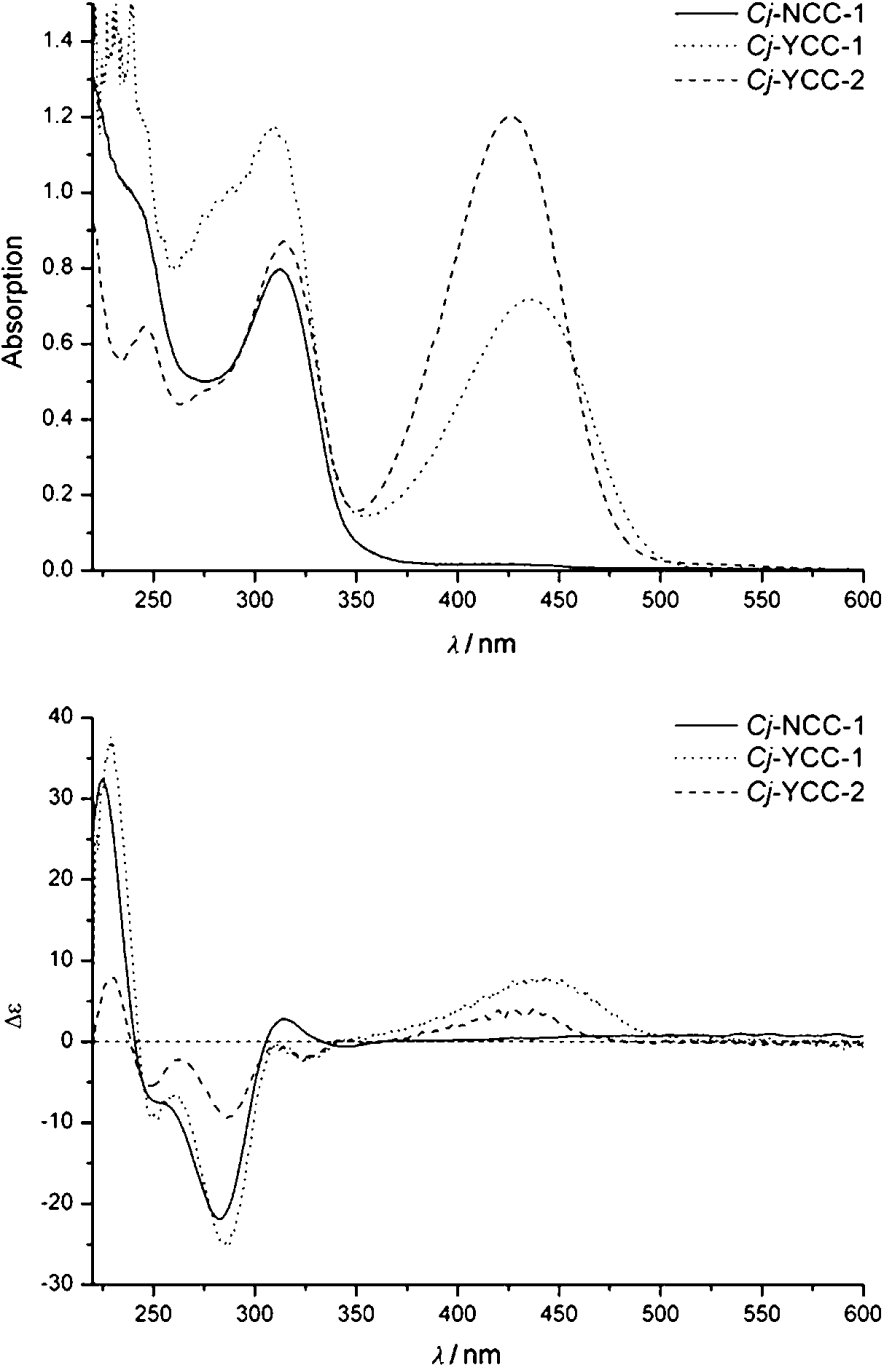
UV/Vis spectra (top) and CD spectra (bottom) of solutions of 40.4 μm *Cj*-NCC-1 (**1**), 28.0 μM *Cj*-YCC-1 (**2 b**) and 37.4 μM *Cj*-YCC-2 (**2 a**) in MeOH.

*Cj*-YCC-1 (**2 b**), the more polar of the two yellow pigments, likewise exhibited a UV/Vis spectrum with two characteristic absorbance bands, with maxima at 313 and 440 nm (relative intensities of 1.00 to 0.61, see Figure 2). The molecular formula of *Cj*-YCC-1 (**2 b**) was deduced as C_35_H_38_N_4_O_8_ from the pseudo-molecular ion at *m*/*z* 643.2. This indicated the yellow tetrapyrrole **2 b** to be an isomer of **2 a**, and to have two H atoms less per molecule than the parent **1**. The constitution of the yellow catabolite *Cj*-YCC-1 (**2 b**) was deduced from ^1^H NMR spectra, as well as homo- and heteronuclear 2-D spectra (ROESY, ^1^H,^13^C-HSQC and HMBC, see Figure 3 and Figure S2 in the Supporting Information): In the ^1^H NMR spectra of **2 b** (CD_3_OD, at 25 °C) the signals of all 34 carbon-bound hydrogen atoms could be observed. Among these, two singlets at lower field, of HC5=O and of HC20, the spin system for a peripheral vinyl group at an intermediate field, a singlet near *δ*=3.7 ppm (ester methyl) and the singlets of four methyl groups at high field stand out. From ^1^H,^13^C heteronuclear NMR correlations (HSQC, g-HMBC and ROESY spectra[10]) of **2 b**, complete assignment of the ^1^H and ^13^C signals could be achieved. The constitution of **2 b** could thus be confirmed as that of an oxidation product of *Cj*-NCC-1 (**1**), resulting from removal of two hydrogen atoms from the C1 and C20 positions (of **1**). ^1^H-ROESY spectra helped to establish the *E* configuration in **2 b** of the new double bond between C20 and C1. On this basis, and assuming that the stereostructure of **2 b** would correspond to that of *Cj*-NCC-1 (**1**) elsewhere, the yellow oxidation product **2 b** was thus assigned the structure of a (13^2^*S*,15*R*,20*E*)-3^1^,3^2^-didehydro-4,5,10,15-(22,24 *H*)hexahydro-13^2^-methoxycarbonyl-4,5-*seco*-4,5-dioxophytoporphyrinate (see Figure 2 and Scheme 2). The suggested retention of the configuration at the C13^2^ and C15 positions was supported by comparison of the basic sign-properties of the CD spectra of **1**, **2 a** and **2 b**. The two isomeric yellow compounds (**2 a** and **2 b**) are thus oxidation products of **1**, from which they both arise by formal loss of two H atoms from the C1 and C20 positions.

**Figure 3:**
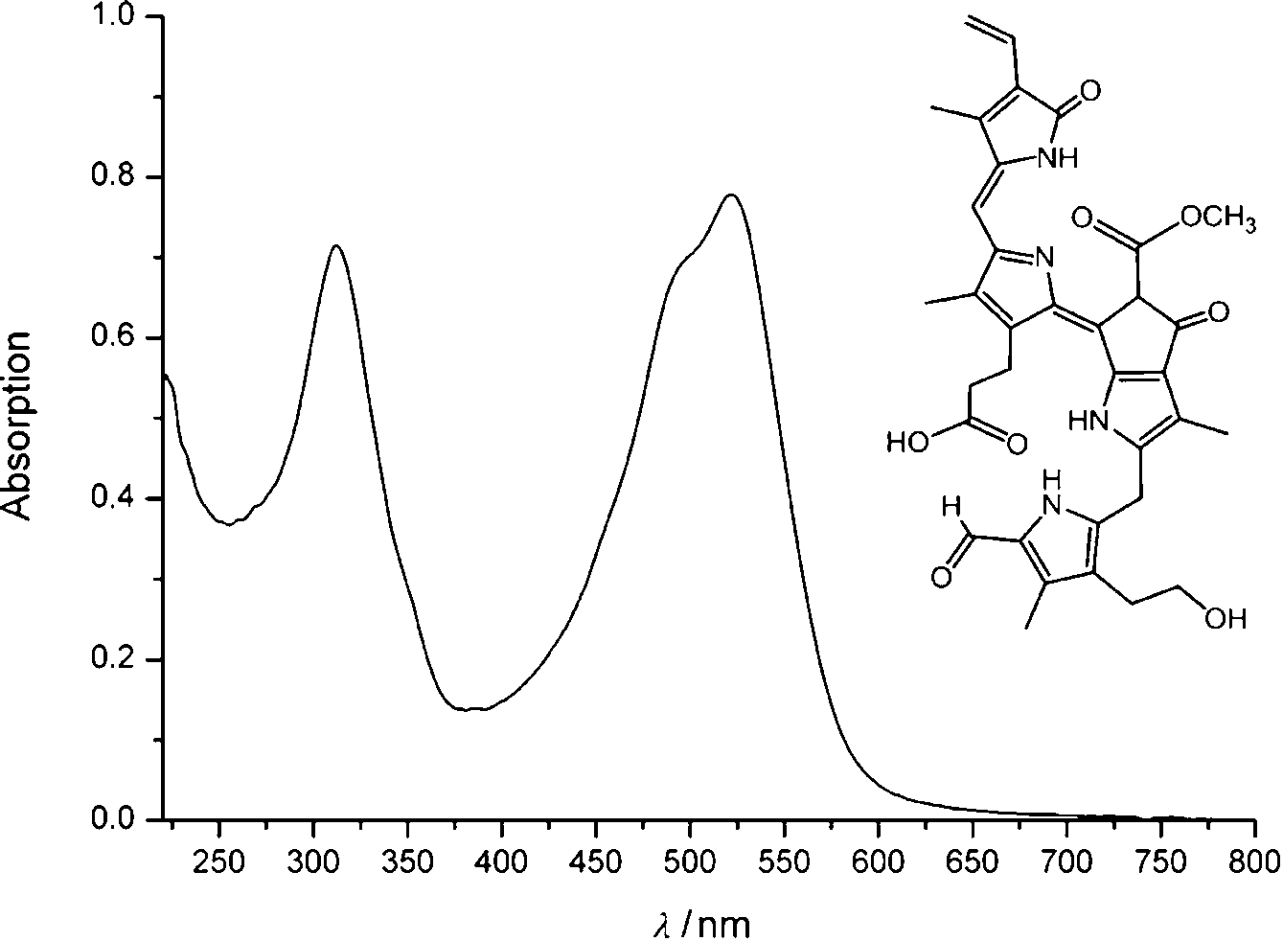
UV/Vis spectrum of a solution of *Cj*-PiCC (**3**, 38.4 μM) in MeOH.

**Scheme 2:**
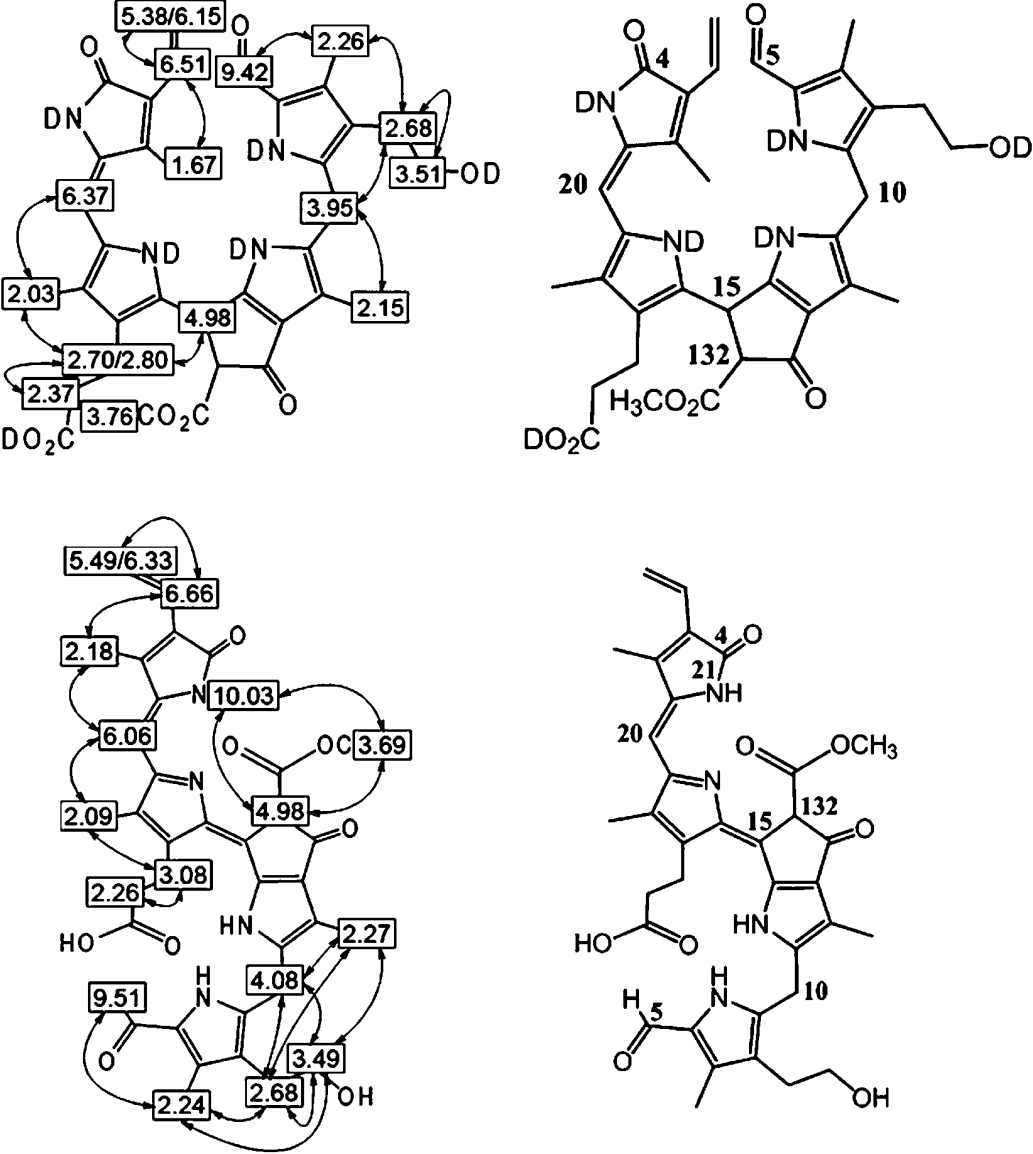
Deduced constitutions of *Cj*-YCC-1 (**2 b**, top), and of *Cj*-PiCC (**3**, bottom): Left: Graphical representations of ^1^H-chemical shift data (500 MHz spectra) with 2D ROESY correlations. Right: Constitutional formulae of **2 b** and **3**. Atom numbering, as recommended by IUPAC for chlorophyll derivatives, see fore example, reference [11].

The significantly less polar pink-red compound *Cj*-PiCC (**3**) was indicated to be a further oxidation product of the YCCs (**2 a**, **2 b**). Its UV/Vis spectrum in MeOH exhibited strong absorbance bands at 312 and 522 nm, with relative intensities of 0.92 to 1.00 (see Figure 3). The molecular formula of **3** was deduced by mass spectrometry to be C_35_H_36_N_4_O_8_, indicating two H atoms less (per molecule) than were present in **2 a** or **2 b**. The constitution of the pink-red catabolite **3** was deduced from ^1^H NMR spectra, as well as homo- and heteronuclear 2D spectra (ROESY, ^1^H,^13^C-HSQC and HMBC,[10] see Scheme 2 and Figure S3 in the Supporting Information): In the ^1^H NMR spectra of *Cj*-PiCC (**3**, in CD_3_CN, at 25 °C) the signals of all 30 carbon-bound hydrogen atoms could be observed. Among these were two singlets at low and at an intermediate field, of HC5=O and of HC20, the spin system for a peripheral vinyl group and the singlets of four methyl groups at high field and of an ester methyl group (near *δ*=3.7 ppm). In addition, the signal of HN21 could be observed. From ^1^H,^13^C heteronuclear NMR correlations (HSQC, g-HMBC) and ROESY spectra of **3**, the complete assignment of the ^1^H and ^13^C signals could be achieved and **3** was delineated to have the constitution of a 3^1^,3^2^-didehydro-4,5,10-(22 *H*)-tetrahydro-13^2^-methoxycarbonyl-4,5-*seco*-4,5-dioxophytoporphyrinate (see Scheme 2). ^1^H-ROESY spectra of **3** helped to establish the *Z* configuration of the double bond between C20 and C1: the observed NOEs (e.g. from the ester methyl group to HN21 of ring A) were all consistent with an *E* configuration of the “new” double bond between C15 and C16. A CD-spectrum of the isolated sample of **3** showed very week signals only. Apparently, practically racemic **3** was isolated, due to equilibration at its single stereo-center, the exchange labile C13^2^. The pink-red oxidation product **3** was thus assigned the structure of a (15*E*,20*Z*)-3^1^,3^2^-didehydro-4,5,10-(22 *H*)-tetrahydro-13^2^-methoxy-carbonyl-4,5-*seco*-4,5-dioxophytoporphyrinate.

Our experiments showed the “rust” colour of the NCCs to develop from oxidative decomposition of the colourless NCCs mainly, as suspected earlier[2] (see Scheme 3). NCCs, the colourless linear tetrapyrroles from breakdown of chlorophyll were revealed to be rather strong antioxidants,[12] nearly as effective as bilirubin.[13] Oxidation of the colourless *Cj*-NCC-1 (**1**) with DDQ provided YCC **2 a**, by dehydrogenation at the C20 *meso* bridge. In YCCs electronic conjugation via the “western” C20 *meso* position extends the tetrapyrrolic chromophore into the visible range.[7] Indeed, YCCs (**2 a** and **2 b**) have a chromophore that is remarkably similar to that of the heme breakdown product bilirubin.[14] Likewise similar to bilirubin (which undergoes light-induced double bond (*Z*–*E*) isomerisations[14]), YCCs **2 a** and **2 b** were observed in analytical experiments to interconvert in solution (e.g. in MeCl_2_), when they are exposed to daylight (see Figure S4 in the Supporting Information). Photo-isomerisation of **2 a** is thus a path for its preparative isomerisation to **2 b**. Further oxidation of the YCCs occurs at the saturated C15 *meso* position, and the extension of the conjugated system via C15 results in the red-shifted absorption properties of *Cj*-PiCC (**3**). This pink-red tetrapyrrole was also prepared in about 40 % yield by direct oxidation of the YCC **2 a** with dichlorodicyanobenzoquinone (DDQ).

**Scheme 3:**
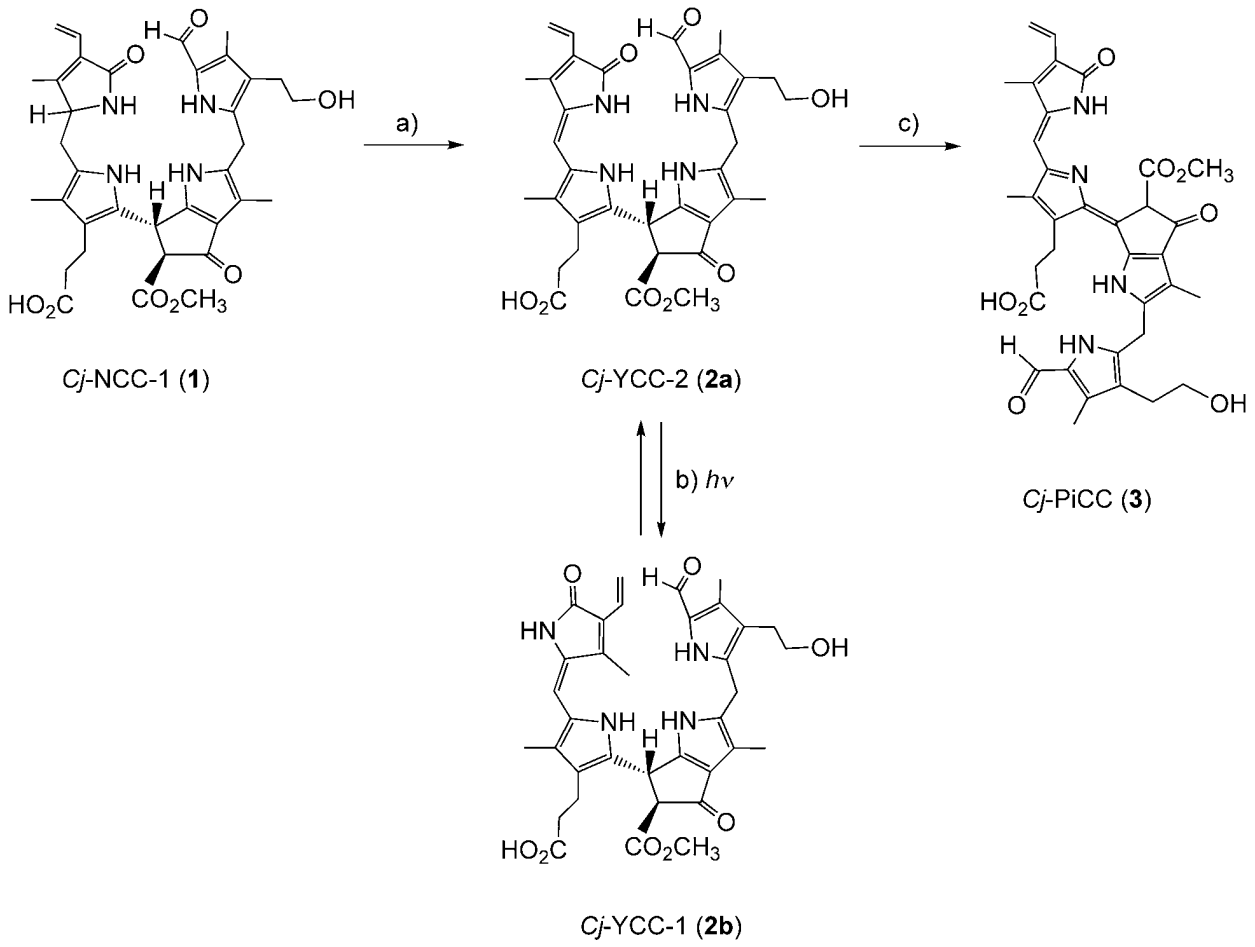
Structural outline of the oxidative decomposition of *Cj*-NCC-1 (**1**). a) Oxidation of **1** to *Cj*-YCC-2 (**2 a**), b) reversible photo-isomerization of **2 a** and **2 b** (*Cj*-YCC-1), c) oxidation of **2 a** to *Cj*-PiCC (**3**).

The ubiquitous colourless NCCs were suggested to represent the “final stage” of chlorophyll breakdown in senescent leaves (see Scheme 1) and were found to accumulate in the vacuoles.[2, 5] Until recently, NCCs were primarily looked at as products of a crucial detoxification process.[15] However, NCCs were recently shown to be effective antioxidants, and they were suggested to possibly play a (still unknown) physiological role in senescent leaves and fruit.[12] Indeed, a compound identified with the YCC **2 a** was observed in small quantities in fresh extracts of senescent leaves of *C. japonicum*.[7, 16] In the leaf, NCC **1** thus appears to be oxidized (by non-enzymic processes) to the yellow YCC **2 a**, from which the isomer **2 b** may possibly be produced by light-induced isomerisation. Further oxidation may result in the pink-red PiCC **3**. A related oxidation process has been proposed to be responsible for the observation of colourless urobilinogenoidic tetrapyrroles in senescent barley leaves,[17] which were suggested to be the result of an alternative oxidation of the *Hv*-NCC-1, the main NCC from de-greened leaves of barley.[4]

Having identified the yellow tetrapyrroles **2 a**/**2 b** and the red-pink compound **3** as oxidation products of the NCC **1**, we have now set out to analyse de-greened plant material (from leaves and ripe fruit) more thoroughly for the appearance of coloured chlorophyll catabolites under the conditions of natural senescence and ripening. Indeed, ongoing studies in our laboratories hint at a more general significance in senescent leaves, not only of yellow chlorophyll breakdown products,[7] but also of their red-pink oxidation products.[18] Red and yellow chlorophyll catabolites may thus prove to be active contributors, after all, to the appealing yellow, orange and red colours of fall leaves.[19]

Coloured tetrapyrroles from chlorophyll breakdown may be of interest as a new class of nature-derived pigments. The yellow and red chlorophyll breakdown products are pigments related to important heme-derived tetrapyrroles, such as biliverdine and its natural reduction products, such as bilirubin and phytochromobilin.[14, 20] Together with the fascinating ‘hypermodified’ fluorescent chlorophyll catabolites (*h*FCCs), that give ripe bananas an intriguing blue luminescence,[21, 22] YCCs and PiCCs represent noteworthy expansions of Nature’s repertoire of plant pigments. In view of the important biological roles played by the heme-derived linear tetrapyrroles, for example, as chromophores in light-sensing enzymes and light-harvesting assemblies in photosynthetic organisms,[20] possible physiological effects of the tetrapyrrolic chlorophyll catabolites in plants and in higher animals continue to call for attention.[21]

## Experimental Section

**General and chromatography**: See details in Supporting Information.

**Spectroscopy**: UV/VIS: HITACHI U-3000 spectrophotometer; *λ*_max_ in nm (rel. ɛ). CD: JASCO J-715 spectropolarimeter; *λ*_max_ and λ_min_ in nm (relative Δɛ). ^1^H NMR: Varian UNITYplus 500; *δ* in ppm with *δ*(CHD_2_OD)=3.39 ppm; *J*_HH_(Hz). MS: MAT 95 sector field instrument with fast atom bombardment (FAB) or electrospray ionisation (ESI) source, positive-ion modes, *m*/*z* (rel. int. in %); FAB (caesium ions at 20 keV, 2 μA): glycerine matrix,. ESI: flow rate 2 mL min^−1^, spray voltage 3.0 kV, solvent water/MeOH 1:1 (v/v).

**Preparative oxidation of** ***Cj*****-NCC-1 (1) to coloured tetrapyrrolic compounds on silica gel**: A sample of NCC **1** (192.9 mg, 270 μmol) was dissolved in MeCl_2_ (150 mL) and silica gel (19.3 g) in dichloromethane (200 mL) was added. The resulting suspension was filtered and dried. The dry powder was stirred magnetically under air, and was illuminated with a 100-W tungsten lamp for 14 h. An orange-red powder was obtained. Analytical HPLC[7] revealed three coloured fractions: yellow *Cj*-YCC-1 (**2 b**, *r*_t_=15 min), yellow *Cj*-YCC-2 (**2 a**, *r*_t_=22 min) and pink-red *Cj*-PiCC (**3**, *r*_t_=35 min). Extraction of the coloured powder and work-up with MPLC (as described[7]) resulted in re-isolated starting material *Cj-*NCC-1 (**1**, 33.5 mg=52.0 μmol=17.4 %) and two yellow pigments, isolated as powders (29.2 mg of *Cj*-YCC-1 (**2 b**, 45.4 μmol=15.2 %) and 30.3 mg of *Cj*-YCC-2 (**2 a**, 47.2 μmol=15.8 %)).[7] A third (red) fraction was also collected and desalted on a Sep-Pak cartridge. Crude pink-red *Cj-*PiCC (**3**) was eluted with CH_3_OH, the solvent was evaporated under reduced pressure and the red residue was re-dissolved in CH_3_OH/water 1:1 (v/v) (2 mL), to be resubmitted to semi-preparative HPLC. After separation, desalting, isolation and drying *Cj*-PiCC (**3**, 5.5 mg, 8.5 μmol, 2.9 %) was obtained as a red powder, which was characterised, as described below.

**Selected spectroscopic data**: ***Cj-YCC-1 (2 b)**:* UV/Vis: (CH_3_OH, *c*=2.80×10^−5^ M): *λ*_max_ (log ɛ)=247 (4.58), 313 (4.62), 440 nm (4.41). CD: (methanol, *c*=2.80×10^−5^ M): *λ*_min/max_ (Δ*ɛ*)=229 (37.7), 251 (−9.7), 261 (−6.5), 286 (−25.1), 312 (−0.3), 326 (−2.7), 442 nm (8.1); ^1^H NMR: (500 MHz, CD_3_OD): *δ*=1.67 (s, H_3_C2^1^), 2.03 (s, H_3_C18^1^), 2.15 (s, H_3_C12^1^), 2.26 (s, H_3_C7^1^), 2.37 (m, H_2_C17^2^), 2.68 (m, H_2_C8^1^), superimposed by 2.70 (m, H_A_C17^1^), 2.80 (m, H_B_C17^1^), 3.51 (m, H_2_C8^2^), 3.76 (s, H_3_C13^5^), 3.95 (s, H_2_C10), 4.98 (s, HC15), 5.38 (dd, *J*=2.5/11.5 Hz, H_A_C3^2^), 6.15 (dd br., *J*=2.5/18 Hz, H_B_C3^2^), 6.37 (s, HC20), 6.51 (dd, *J*=12/17.5 Hz, HC3^1^), 9.42 ppm (s, HC5); MS (ESI positive-ion mode, CH_3_OH/H_2_O 1:1 (v/v), spray voltage 3.0 kV): *m/z* (%): 643.20 (15, [*M*+H]^+^), 665.10 (35, [*M*+Na]^+^), 681.13 (100, [*M*+K]^+^), 719.04 (20, [*M*−H+2K)^+^], 1307.58 (5, [2*M*+Na]^+^), 1323.49 (10, [2*M*+K]^+^).

***Cj-YCC-2 (2 a)***: UV/Vis (CH_3_OH, *c*=3.74×10^−5^ M): *λ*_max_ (log *ɛ*)=244 (4.22), 310 (4.35), 426 nm (4.51); CD (CH_3_OH, *c*=3.74×10^−5^ M): *λ*_min/max_ (Δ*ɛ*)=229 (7.9), 248 (−5.6), 263 (−2.2), 287 (−9.2), 311 (−1.1), 325 (−1.8), 345 (0.2), 357 (−0.2), 429 nm (3.6).

***Cj-PiCC (3)***: UV/Vis (CH_3_OH, *c*=4.69×10^−5^ M): *λ*_max_ (log *ɛ*)=312 (4.27), 495 (4.25), 522 nm (4.31); ^1^H NMR (500 MHz, CD_3_OD): *δ*=2.12 (s, H_3_C12^1^), 2.14 (s, H_3_C18^1^), 2.22 (s, H_3_C2^1^), 2.29 (s, H_3_C7^1^), 2.42 (m, H_2_C17^2^), 2.69 (m, H_2_C8^1^), 3.13 (m, H_2_C17^1^), 3.50 (m, H_2_C8^2^), 3.76 (s, H_3_C13^5^), 4.24 (s, H_2_C10), 5.50 (dd, *J*=2.2/12.0 Hz, H_A_C3^2^), 6.14 (HC20), 6.33 (dd, *J*=2.2/17.6 Hz, H_B_C3^2^), 6.65 (dd, *J*=12.0/17.6 Hz, HC3^1^), 9.45 ppm (s, HC5); MS (HR-FAB, pos.): *m/z* 641.2626 (exptl): *m*/*z* 641.2606 (calcd) [*M*+H]^+^ C_35_H_37_O_8_N_4_.
