# Supplementary material for: How the Colourless ‘Nonfluorescent’ Chlorophyll Catabolites Rust
Source: Chemistry. 2011 Feb 18;17(8):2330–4. doi: 10.1002/chem.201003313 (PMC3072522; doi:10.1002/chem.201003313)
Supplement: Supplementary file 1 [file chem0017-2330-SD1.pdf]

CHEMISTRY
A EUROPEAN JOURNAL

Supporting Information

© Copyright Wiley-VCH Verlag GmbH & Co. KGaA, 69451 Weinheim, 2011

How the Colourless 'Nonfluorescent' Chlorophyll Catabolites Rust

Markus Ulrich, Simone Moser, Thomas Müller, and Bernhard Kräutler*[a]

chem_201003313_sm_miscellaneous_information.pdf

Experimental Part.

General. Solvents for extractions were regent-grade commercials and distilled before use. HPLC grade methanol (MeOH) was from Merck (Darmstadt, Germany) and Acros Organics (Geel, Belgium). Dichloromethane ($MeCl_2$), potassium dihydrogen phosphate puriss p.a., potassium phosphate dibasicanhydrous puriss.p.a. and 2,3-Dichloro-5,6-dicyano-1,4-benzoquinone purum (DDQ) were from Fluka (Buchs, Switzerland). Hexane pure was from Acros Organics (Geel, Belgium). Samples of *Cj*-NCC-1 (**1**) and *Cj*-YCC-2 (**2a**) were prepared as described earlier. [S1] Sep-Pak-C18 Cartridges were obtained from Waters Associates. pH values were measured with a WTW Sentix 21 electrode connected to a WTW pH535 digital pH meter.

Spectroscopy. UV/VIS: HITACHI U-3000 spectrophotometer; λ_{max} in nm (rel. ε). CD: JASCO J-715 spectropolarimeter; λ_{max} and λ_{min} in nm (relative Δε). ^{1}H-NMR: Varian UNITYplus 500; δ in ppm with $\delta(CHD_2OD)$ = 3.39 ppm and $\delta(CHD_2CN)$ = 1.94 ppm; J_{HH}(Hz). ^{13}C-NMR (125 MHz, Varian UNITYplus 500): signal assignment from ^{1}H,^{13}C-heteronuclear HSQC and HMBC experiments [S2]. MS: MAT 95 double focusing sector field instrument with fast atom bombardment (FAB) or electrospray ionization (ESI) source; FAB: positive ion mode, (caesium ions at 20 keV, 2 µA), matrix: glycerine, m/z (rel. int. in %). ESI: pos. ions, flow rate 2 mlmin^{-1}, spray voltage 3.0 kV, solvent water/MeOH 1:1 (v/v).

Chromatography. TLC. Silica gel plates POLYGRAM Sil G / UV_{254}, Macherey-Nagel (Düren, Germany). HPLC. solvents: solvent A = 100 mM potassium phosphate buffer (pH 7.0), solvent B = MeOH, C = water. Analytical HPLC (injection volume: 100 µl or as specified in the text) 250 x 4.6 mm column, flow rate 0.5 ml.min^{-1}. Solvent composition: 0-3 min: A : B 80:20 (v/v); 3-7 min: constant gradient of A : B from 80:20 to 40:60 (v/v); 7-25 min: A : B 40:60 (v/v); 25-27 min: linear change from A : B 40:60 to B : C 60:40 (v/v); 27-31 min: linear change from B : C 60:40 to 100:0 (v/v); 31-41 min: B : C 100:0 (v/v); 41-49 min: linear change from B : C 100:0 to 20:80 (v/v); 49-53 min: linear change from B : C 20:80 to A : B 80:20 (v/v). Semi-preparative HPLC (2 ml injection volume, 21.6 mm column, flow rate 5 ml.min^{-1}), solvent composition: 0-10 min: A : B 80:20 (v/v); 10-40 min: constant gradient of A : B from 80:20 to 40:60 (v/v). MPLC, as described. [S1]

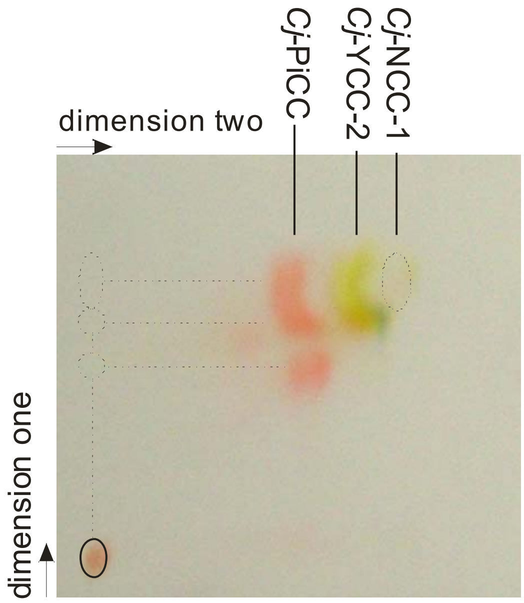

Figure S1: 2-D TLC analysis of the rust formation from the NCC **1**: 0.1 mg *Cj*-NCC-1 was dissolved in 1 ml of MeOH. A sample of 18 µl was spotted on a TLC plate (4 x 4 cm), which was stored for 2 minutes at room temperature and at day light. An analytical trace was developed in one dimension (solvent composition: $CHCl_3$/MeOH 85/15 (v/v)). The TLC plate was exposed to day light for 3 minutes. The plate then was developed in the second dimension (same solvent composition).

Synthesis of Cj-PiCC by DDQ-oxidation of Cj-YCC-2. 25.5 mg (09.7 µmol) of a *Cj*-YCC-2 (**2a**) sample were dissolved in 12 ml of acetone. 8.8 mg of DDQ (1 molequivalent) were dissolved in 5 ml acetone. Both solutions were first flushed with argon for 1 minute and cooled in an N_2(l)/ethanol bath to –45°C. The DDQ solution was directly injected by a syringe into *Cj*-YCC-2 (**2a**) solution. The deep red reaction mixture was stirred in the freezing mixture under argon for 2 h; during this time the temperature gradually increased to –21.1 °C. The reaction mixture was diluted with 400 ml 100 mM potassium phosphate buffer (pH 7.0) and was desalted by using a SepPak cartridge. The products were eluted with 50 ml CH_3OH and the sample was dried to give 25.1 mg of raw product.

The raw sample was dissolved by 20 ml CH_3OH and diluted with 80 ml 100 mM potassium phosphate buffer (pH 7.0). The deep red solution was applied to an MPLC system. The fractions containing *Cj*-PiCC (**3**) were collected and their volume was reduced to 40 %, using a rotary evaporator. The remaining mixture was diluted with an equal amount of water and applied to a SepPak cartridge. It was first desalted by washing with water and the red fraction of **3** was then eluted with 50 ml of methanol. The solvent was evaporated and the residue was dried in vacuo to give 9.2 mg (36.2 % yield) of *Cj*-PiCC (**3**).

Spectroscopic data.

***Cj*-YCC-1** (**2b**). ^{1}H-NMR: (500 MHz, CD_3OD, see Figure S2): δ [ppm] = 1.67 (s, ($H_3C(2^1)$), 2.03 (s, $H_3C(12^1)$), 2.15 (s, $H_3C(18^1)$), 2.26 (s, $H_3C(7^1)$), 2.37 (m, $H_2C(17^2)$), 2.68 (m, $H_2C(8^1)$), superimposed by 2.70 (m, $H_AC(17^1)$), 2.80 (m, $H_BC(17^1)$), 3.51 (m, $H_2C(8^2)$), 3.76 (s, $H_3C(13^5)$), 3.95 (s, $H_2C(10)$), 4.98 (s, HC(15)), 5.38 (dd, J = 2.5/11.5 Hz, $H_AC(3^2)$), 6.15 (dd broad, J = 2.5/18 Hz, $H_BC(3^2)$), 6.51 (dd, J = 12/17.5 Hz, $HC(3^1)$), 9.42 (s, HC(5)). ^{13}C-NMR: (125 MHz, CD_3OD, see Figure S2): δ[ppm] = 9.29 (12^1), 9.39 (7^1), 9.59 (18^1), 12.2 (2^1), 22.1 (17^1), 23.5 (10), 27.8 (8^1), 37.0 (15), 40.1 (17^2), 52.1 (13^5), 62.6 (8^2), 107.9 (20), 112.5 (12), 119.3 (3^2), 121.1 (8), 123.2 (17), 124.0 (13), 124.7 (19), 127.0 (3^1), 129.4 (16), 129.6 (3), 129.6 (6), 134.6 (11), 137.1 (1), 138.4 (9), 140.5 (2), 161.4 (14), 171.6 (13^3), 182.3 (17^3).

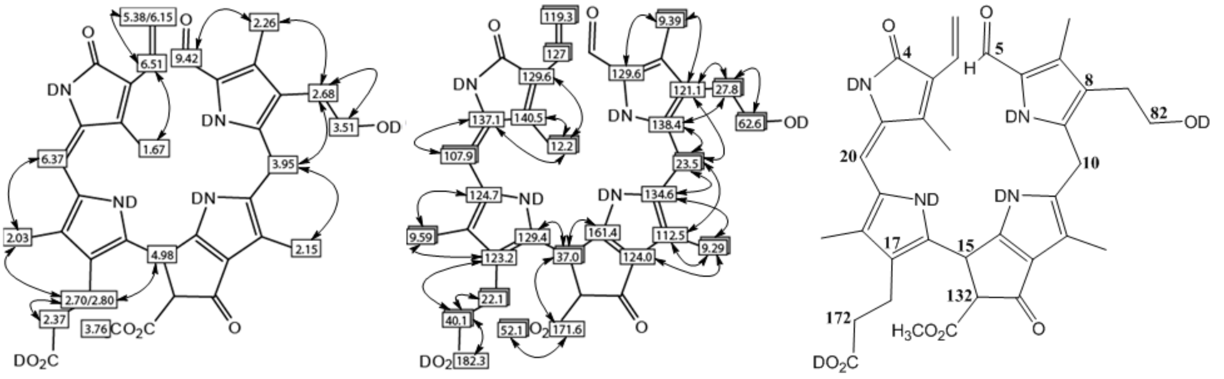

Figure S2: Graphical representations of ^{1}H- and ^{13}C chemical shift data (500 MHz spectra, in CD_3OD), 2D-correlations and deduced constitutional formula of *Cj*-YCC-1 (**2b**). Left: ROESY-correlations; centre: assignments due to ^{1}J correlations from ^{1}H-^{13}C-HSQC spectra (shadowed boxes), and from ^{1}H-^{13}C-long range couplings from HMBC-spectra, arrows, see e.g. [S2]); right: constitutional formula of *Cj*-YCC-1 (**2b**). Atom numbering, as recommended by IUPAC for chlorophyll derivatives, see e.g.[S3].

***Cj*-PiCC** (**3**): ^{1}H-NMR (500 MHz, CD_3OD): δ [ppm] = 2.12 (s, $H_3C(12^1)$), 2.14 (s, $H_3C(18^1)$), 2.22 (s, $H_3C(2^1)$), 2.29 (s, $H_3C(7^1)$), 2.42 (m, $H_2C(17^2)$), 2.69 (m, $H_2C(8^1)$), 3.13 (m, $H_2C(17^1)$), 3.50 (m, $H_2C(8^2)$), 3.76 (s, $H_3C(13^5)$), 4.24 (s, $H_2C(10)$), 5.50 (dd, J = 2.2/12.0 Hz, $H_AC(3^2)$), 6.14 (HC(20)), 6.33 (dd, J = 2.2/17.6 Hz, $H_BC(3^2)$), 6.65 (dd, J = 12.0/17.6 Hz, $HC(3^1)$), 9.45 (s, HC(5)). ^{13}C-NMR (125 MHz, CD_3OD): δ[ppm] = 7.98 (2^1), 7.75 (7^1), 8.27 (18^1), 22.9 (10), 26.7 (17^2), 26.8 (8^1), 52.0 (13^5), 62.8 (8^2), 98.4 (20), 115.3 (12), 120.1 (8), 126.0 (3^2), 128.6 (1), 127.9 (3^1), 124.2 (17), 128.5 (6), 136.3 (7), 136.5 (11), 137.0 (19), 141.3 (2), 144.3 (9).

^{1}H-NMR: (500 MHz, CD_3CN, see Figure S3): δ [ppm] = 2.09 (s, $H_3C(18^1)$), 2.18 (s, $H_3C(2^1)$), 2.24 (s, $H_3C(7^1)$), 2.26 ($H_2C(17^2)$), 2.27 (s, $H_3C(12^1)$), 2.68 ($H_2C(8^1)$), 3.08 ($H_2C(17^1)$), 3.49 ($H_2C(8^2)$), 3.69 (s, $H_3C(13^5)$), 4.08 (s, $H_2C(10)$), 4.98 (s, $HC(13^2)$), 5.49 (dd, J = 2.2/11.7 Hz, $H_AC(3^2)$), 6.06 (HC(20)), 6.33 (dd, J = 2.2/17.6 Hz, $H_BC(3^2)$), 6.66 (dd, J = 11.7/17.6 Hz, $HC(3^1)$), 9.51 (HC(5)), 10.03 (HN(21)), 12.3 ($HOOC(17^3)$). ^{13}C-NMR: (125 MHz, CD_3CN, see Figure S3): δ [ppm] = 8.74 (7^1), 9.48 (2^1), 9.48 (12^1), 9.48 (18^1), 23.5 (10), 25.1 (17^1),

28.4 (8^1), 40.6 (17^2), 52.3 (13^5), 62.8 (8^2), 66.9 (13^2), 98.9 (20), 115.8 (12), 120.6 (8), 121.1 (3^2), 121.2 (3), 127.6 (3^1), 128.2 (1), 129.5 (6), 129.5 (14), 135.9 (7), 136.7 (11), 137.8 (15), 138.4 (18), 142.1 (2), 142.4 (17), 144.2 (9), 146.3 (13), 147.4 (16), 166.5 (19), 170.6 (13^3), 171.2 (4), 188.1 (13^1)

MS (ESI pos., CH_3OH/H_2O 1:1 (v/v), spray voltage 1.2 kV): m/z (%) = 456.06 (25, $[M\text{-}CH_3OH\text{-ring B+H}]^+$), 488.13 (15 $[M\text{-ring B+H}]^+$), 609.09 (15, $[M\text{-}CH_3OH\text{+H}]^+$), 641.12 (100, $[M+H]^+$), 663.25 (25, $[M+Na]^+$), 686.09 (15, $[M\text{-}H+2Na]^+$), 701.12 (20, $[M\text{-}H+Na+K)^+$].

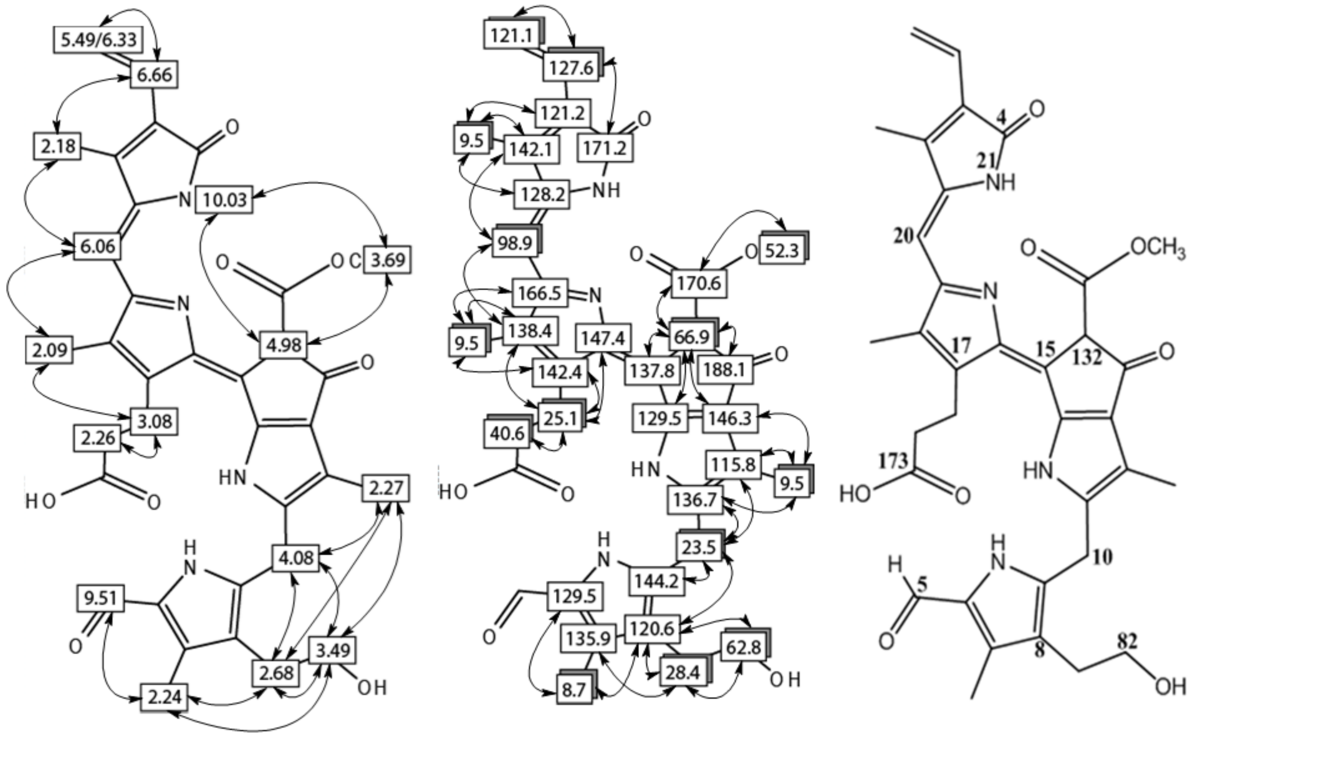

Figure S3: Graphical representations of ^1H- and ^{13}C chemical shift data (500 MHz spectra, in CD_3CN), 2D-correlations and deduced constitutional formula of *Cj*-PiCC (**3**). Left: ROESY-correlations; centre: assignments due to ^1J correlations from ^1H-^{13}C-HSQC spectra (shadowed boxes), and from ^1H-^{13}C-long range couplings, from HMBC-spectra, arrows, see e.g. [S2]), right. constitutional formula of *Cj*-PiCC (**3**). Atom numbering, as recommended by IUPAC for chlorophyll derivatives, see e.g. [S3].

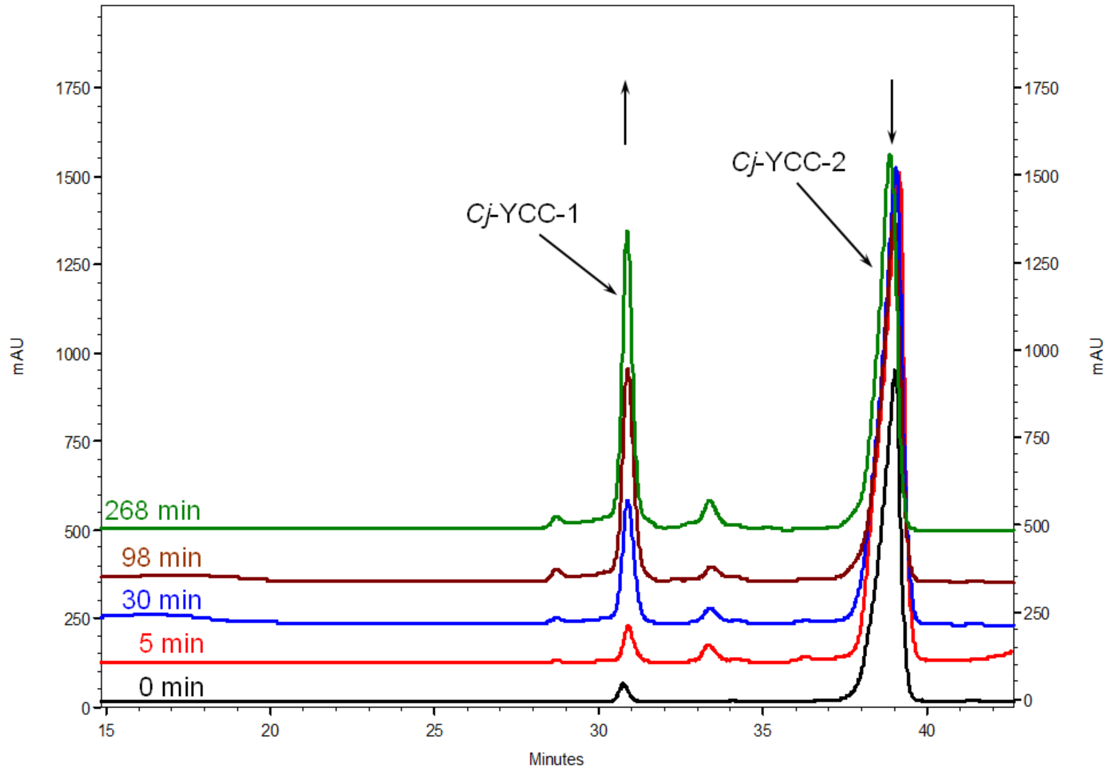

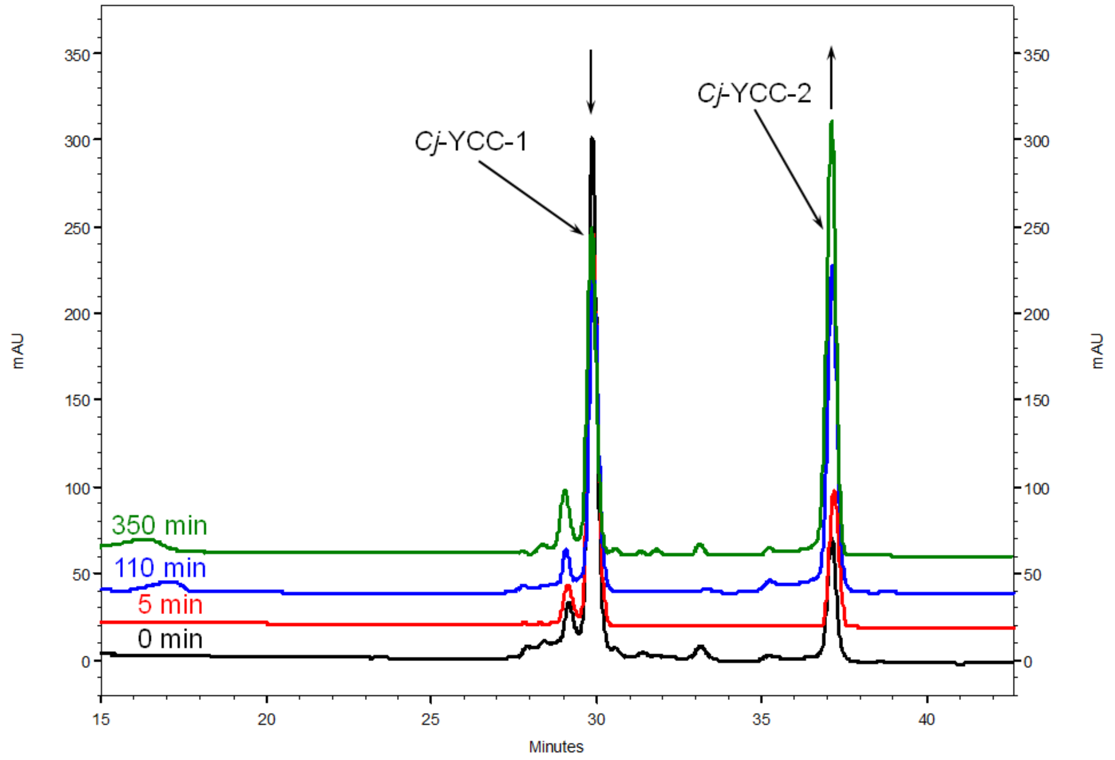

Figure S4: Analytical investigations of the photo-isomerization of YCCs by HPLC. Samples of *Cj*-YCC-2 (**2a**), at top, and of *Cj*-YCC-1 (**2b**), at bottom, were dissolved in MeOH / $MeCl_2$, (1:15), the solutions were purged with Ar and then exposed to irradiation with day light at room temperature; samples were taken at indicated times; analytical HPLC, optical detection at 320 nm. Top. *Cj*-YCC-2 (**2a**) is partially transformed into *Cj*-YCC-1 (**2b**); bottom. *Cj*-YCC-1 (**2b**) is partially transformed into *Cj*-YCC-2 (**2a**).

*Preparative photoisomerization of Cj-YCC-2 (**2a**).* A sample of 38.6 mg *Cj*-YCC-2 (**2a**, 60.1 µmol) was dissolved in 2 ml MeOH and 10 ml acid free $MeCl_2$, and the solution was flushed with argon for 1 min. The deep yellow solution was stirred at r.t. and in the presence of day light for a total of 22 h. The solvent was evaporated in vacuo, to give 38.2 mg of a raw yellow residue. This was dissolved in 6 ml MeOH and 28 ml 100 mM potassium phosphate buffer (pH 7.0) were added. The orange solution was applied to the MPLC system. The fractions containing *Cj*-YCC-1 and *Cj*-YCC-2 were collected and partially concentrated to about 40% volume under reduced pressure. For desalting, they were then separately applied to a SepPak cartridge, washed with water and eluted with 50 ml MeOH. The solvent was evaporated and the residues were dried in vacuo to give 8.7 mg *Cj*-YCC-1 (**2b**, 13.5 µmol = 22.5% yield) and 22.4 mg *Cj*-YCC-2 (**2a**, 34.9 µmol = 58.1% yield).

References

[S1] S. Moser, M. Ulrich, T. Müller and B. Kräutler, *Photochem. Photobiol. Sci.* **2008**, *7*, 1577-1581.
[S2] H. Kessler, M. Gehrke and C. Griesinger, *Angew Chem Int Ed* **1988**, *27*, 490-536.
[S3] H. Scheer in *Chlorophylls*, (Ed. H. Scheer), CRC Press, Boca Raton, USA, **1991**, pp. 3-30.
